# *Listeria monocytogenes* Invasion Into Sheep Kidney Epithelial Cells Depends on InlB, and Invasion Efficiency Is Modulated by Phylogenetically Defined InlB Isoforms

**DOI:** 10.3389/fmicb.2022.825076

**Published:** 2022-02-07

**Authors:** Yaroslava Chalenko, Olga Kolbasova, Elena Pivova, Mariam Abdulkadieva, Olga Povolyaeva, Egor Kalinin, Denis Kolbasov, Svetlana Ermolaeva

**Affiliations:** ^1^Laboratory of Ecology of Pathogenic Bacteria, Gamaleya Research Center of Epidemiology and Microbiology, Moscow, Russia; ^2^Federal Research Center for Virology and Microbiology (FRCVM), Volginsky, Russia; ^3^Department of Dusty Plasma, Joint Institute for High Temperatures, Russian Academy of Sciences, Moscow, Russia; ^4^Federal Research Center for Virology and Microbiology (FRCVM), Nizhny Novgorod Research Veterinary Institute Branch, Nizhny Novgorod, Russia

**Keywords:** *Listeria monocytogenes*, listeriosis, small ruminants, invasion factor, InlB, clonal complex

## Abstract

The facultative intracellular pathogen *Listeria monocytogenes* is of major veterinary importance in small ruminants. Nevertheless, details of *L. monocytogenes* interactions with cells of small ruminants are not fully established. To study the potential of *L. monocytogenes* to infect sheep cells, we used the finite sheep kidney cell line (shKEC), which was infected with the wild-type *L. monocytogenes* strain EGDe. The invasion efficiency was 0.015 ± 0.004%. The invasion factor InlB was critically important for invasion, and inlB gene deletion almost prevented *L. monocytogenes* invasion into shKEC cells. Comparison of the potential of phylogenetically defined InlB isoforms to restore the invasive phenotype of the EGDeΔinlB strain demonstrated that although all InlB isoforms restored invasion of the EGDeΔinlB strain into shKEC cells, the InlB isoforms typical of highly virulent ruminant strains of the clonal complexes CC1 and CC7 were more efficient than isoforms typical of CC2 and CC9 strains (which are less virulent toward ruminants) in supporting invasion. *Listeria monocytogenes* effectively multiplied with a doubling of time in about 90 min after they entered the sheep cells. Intracellular bacteria moved using the well-known actin polymerization mechanism. Cell-to-cell spreading was restricted to the infection of a few tens of neighboring cells for 7 days. Overall, the obtained results demonstrated that (i) InlB is required for invasion into sheep cells, (ii) InlB isoforms might be important for hypervirulence of certain clonal groups toward ruminants, and (iii) *L. monocytogenes* effectively multiplies in ovine cells once entered.

## Introduction

The Gram-positive bacterium *Listeria monocytogenes* causes a serious foodborne disease, listeriosis, in humans and farm animals (Vázquez-Boland et al., 2001; Nightingale et al., 2004b; Allerberger and Wagner, 2010; Walland et al., 2015). Listeriosis is second to salmonellosis as a cause of human death associated with zoonotic foodborne pathogens (Vaillant et al., 2005; Chlebicz and Śliżewska, 2018). In animals, *L. monocytogenes* causes central nervous system infections and abortion, and mortality rates range from 20 to 100% among animals with the developed disease (Miyashita et al., 2004; Nightingale et al., 2004a, 2005; Oevermann et al., 2010; Bundrant et al., 2011; Rocha et al., 2013; Dreyer et al., 2016; European Food Safety Authority (EFSA), 2016; Chlebicz and Śliżewska, 2018; Papić et al., 2019, 2020).

*Listeria monocytogenes* in small ruminants and cattle is of major veterinary importance; average incidence rates of the bacteria range from 5 to 26 cases per 100,000 animals in small ruminants and approximately one case per 100,000 animals in cattle (Papić et al., 2019). Additionally, mass testing showed that approximately 2% of domestic ruminants are Listeria positive in the EU, thus suggesting that asymptomatic carriage is often among ruminants (European Food Safety Authority and European Centre for Disease Prevention and Control (EFSA and ECDC), 2019). Both individual animal resistance and strain virulence are responsible for the selective development of the disease in a part of the infected flock. The *L. monocytogenes* species is divided into four phylogenetic lineages with different virulence potentials (Tsai et al., 2011; Moura et al., 2016; Charlier et al., 2017). Strains of phylogenetic lineages I and II are associated with listeriosis in ruminants (Dreyer et al., 2016; Papić et al., 2019). Lineage I most often causes outbreaks and sporadic cases of listeriosis among humans. Lineages III and IV are very rare, if any are found, among human and animal isolates. The clonal complex CC1 (which belongs to phylogenetic lineage I) is recognized as the most prevalent clonal complex associated with rhombencephalitis in ruminants (Dreyer et al., 2016; Moura et al., 2016; Papić et al., 2019). In addition to CC1, clonal complexes CC4 and CC6 (which belong to lineage I) and CC7, CC37, and CC412 (which belong to lineage II) are described as predominant in ruminant listeriosis cases in Europe (Dreyer et al., 2016; Papić et al., 2019). The ranges of clonal groups, which prevail in listeriosis cases in humans and ruminants, overlap but are not identical (Gray et al., 2004; Tsai et al., 2011). The clonal complexes CC1, CC4, and CC6 are recognized as the most frequent in both human and ruminant CNS and maternofetal infections (Moura et al., 2016; Charlier et al., 2017). On the other hand, the hypervirulent clonal complex CC2 (lineage I), which is described as strongly associated with a clinical origin in humans, has not yet been reported as an important causative agent in ruminants. The clonal complexes CC7 and CC37, which are associated with abortion in ruminants, are described as intermediate virulence clones in humans (Charlier et al., 2017; Papić et al., 2019).

*Listeria monocytogenes* is a facultative intracellular pathogen, and the ability to infect nonprofessional phagocytes is important for its virulence. The hypervirulent clones were shown to be hyperinvasive, and hyperinvasiveness was suggested to be important for virulence (Rupp et al., 2017; Aguilar-Bultet et al., 2018). The protein of InlB internalins is one of two major factors in *L. monocytogenes* invasion into epithelial and endothelial cells (Radoshevich and Cossart, 2018). The tyrosine kinase receptor c-Met is the primary InlB target (Shen et al., 2000). Interactions of InlB with c-Met activate c-Met-controlled signaling pathways that result in actin cytoskeleton rearrangements and bacterial uptake (Gessain et al., 2015; Chalenko et al., 2019). Activation of c-Met by InlB is species-specific, and guinea pig and rabbit cells expressing c-Met do not respond to InlB (Khelef et al., 2006). The complement system receptor gC1q-R, also known as p32, is another InlB target that affects bacterial invasion (Braun et al., 2000; Chalenko et al., 2019). Distinct InlB isoforms were described that differ by lineage-specific and clone-specific substitutions (Moura et al., 2016; Sobyanin et al., 2017b). Some isoforms are more frequent and might occur in strains belonging to distinct clonal complexes (Sobyanin et al., 2017b; Psareva et al., 2019). In human cells, InlB isoforms provide different activation of c-Met-dependent signaling pathways, interactions with gC1q-R, and cell responses (Chalenko et al., 2019). In this work, we studied *L. monocytogenes* interactions with sheep epithelial cells. In particular, we were interested in whether InlB is required for active *L. monocytogenes* invasion into ruminant epithelial cells and whether InlB isoforms could modulate invasion efficiency.

## Materials and Methods

### Cell Line and Cultivation Conditions

The sheep kidney cell line (shKEC) line was obtained at Federal Research Center for Virology and Microbiology (Russia) in 1997 by S. G. Yurkov and N. A. Chermashentseva. shKEC cells were inoculated in the MEM medium (HyClone, Marlborough, MA, United States) with 10% FBS (HyClone, Marlborough, MA, United States) and cultivated into a 24-well plate (Corning, Corning, NY, United States) and incubated at 37°C in a 5% CO_2_ atmosphere.

### qPCR

Genomic DNA was extracted from the sample using a QIAamp® DNA Mini Kit (QIAGEN). The *cytb* gene region was selected for cell identification. The amplification was performed with the following primers and probe: forward primer Oa: 5′-CCT-AAT-CCT-CAC-ATT-CCT-AGT-GGT-AGT-AA-3′, reverse primer Oa: 5′-TAA-TGA-TGT-СGA-GGT-ATT-CAA-CTG-GCT-GG-3′, probe Oa: FAM-CAG-CTG-CGT-ACA-TGA-ATG-CAA-CGG-AGG–BHQ1. To check the DNA extraction and amplification quality, the conserved region of 18S ribosomal DNA was used as an internal control. The amplification was performed with the following primers and probe: forward primer EC: 5′-ACC-CAT-TCG-AAC-GTC-TGC-CCT-3′, reverse primer EC: 5′-CGC-GCC-TGC-TGC-CTT-CCT-3′, probe EC: Cy5-AGC-CGT-TTC-TCA-GGC-TCC-CTC-T–BHQ2. PCR conditions were set as follows: Polymerase activation at 95°C for15 min followed by 45 cycles: 94°C – 20 s, 60°C – 20 s, and 72°C – 20 s.

### Bacterial Strains

The wild-type *L. monocytogenes* strain EGDe and the strain EGDeΔinlB strain with inlB deletion was generously provided by Prof. J. Vazquez-Boland, Univ. Edinburgh, United Kingdom, and have been used in previous studies (Kibardin et al., 2006; Sobyanin et al., 2017a). Complementation of the inlB deletion with the InlB-expressing plasmid was described earlier (Sobyanin et al., 2017b). Briefly, the *inlB* gene was expressed from the promoter of the *inlAB* operon cloned into the shuttle vector pTRKH2 (O’Sullivan and Klaenhammer, 1993; Sobyanin et al., 2017b). The inlB alleles were cloned from strains of clonal complexes CC1, CC2, CC7, and CC9. Corresponding alleles were designated as variant 9, variant 1, variant 14, and variant 13 in Sobyanin et al. (2017b). In this work, the recombinant proteins are designated as InlBCC1, InlBCC2, InlBCC7, and InlBCC9. Recombinant strains expressing a specific variant of internalin B are indicated EGDeΔinlB::InlBCC1, EGDeΔinlB::InlBCC2, EGDeΔinlB::InlBCC7, and EGDeΔinlB::InlBCC9. To maintain the plasmid, erythromycin (Sigma-Aldrich, St. Louis, MO, United States) was added to the medium to a concentration of 10 μg ml^−1^. *Listeria monocytogenes* was cultivated in the BHI medium (Becton, Dickinson and Company, East Rutherford, NJ, United States) and grown at 37°C with agitation at 180 rpm. Plasmid-bearing strains were grown in the presence of 10 μg/ml erythromycin. To prepare a culture for infection, bacteria were grown to the mid-exponential phase, washed with PBS (Amresco, Solon, OH, United States) three times, aliquoted, and frozen in the presence of 10% glycerol (Sigma-Aldrich, St. Louis, MO, United States). The use of pre-frozen cultures allows for more accurate standardization of infection doses between the compared strains. The concentration of bacterial cells was determined by plating serial dilutions from the frozen culture the day before the experiment.

### *In vitro* Invasion and Proliferation Assay

Sheep kidney cell line cells from the fourth passage of subcultures were used in the experiment. Before the analysis of invasion and proliferation, shKEC cells were cultured without antibiotics for 5 days. An invasion assay was performed according to Suárez et al. (2001). Bacteria frozen in 10% glycerol were thawed immediately before the experiment and resuspended in PBS to a volume of 1 ml. The bacterial suspension was diluted in DMEM with 10% FBS to achieve final concentrations with a multiplicity of infection (MOI) of 100 CFU per cell and added to the cells reached 70–80% confluence in a 24-well. After 1 h incubation at 37°C in a 5% CO_2_ atmosphere, cells were washed with PBS three times, and fresh DMEM supplemented with 100 μg ml^−1^ gentamicin (Sigma-Aldrich, St. Louis, MO, United States) was added to kill extracellular bacteria. After 1 h of contact with gentamicin, cells were washed with PBS and added 100 Triton X-100 (Panreac, Barcelona, Catalonia, Spain). Then, 900 μl of PBS was added to each well and serial dilutions were made and sown on solid BHI medium. Plates were incubated at 37°C for 24 h, and then colonies were quantified. The efficiency of invasion was evaluated as a percentage of the number of entered bacteria relatively to that of bacteria used for cell infection. To assess intracellular proliferation, infected cells were incubated in the gentamicin-containing medium at 37°C. Cells were lysed at 2, 4, 6, and 8 hours post-infection (hpi), and bacteria were plated from lysates as described above. The effectiveness of intracellular proliferation was evaluated by determining the number of CFU at this time to the number of introduced bacteria. All the experiments were performed in triplicate and repeated at least three times.

### Plaque Forming Assay

Bacteria for the assay were prepared and used for the infection of the confluent monolayer of shKEC cells cultured in six-well plates. The assay was performed in general as described in Sun et al. (1990) and Sokolovic et al. (1993). About 1 h incubation, the cells were washed three times with PBS, and non-penetrated bacteria were killed by 1 h incubation with gentamicin (100 μg ml^−1^). The infected monolayers were then overlayed with 10 ml of the soft agar, prepared by mixing equal volumes of 2 × DMEM supplemented with 20 μg ml^−1^ gentamicin and 2% agar. Cells were further incubated under 5% CO_2_ at 37°C for up to 3 days following infection, and plaques were visualized by staining monolayers with 1 ml Neutral Red Solution (Sigma Aldrich, St. Louis, MO, United States) diluted 1:10 in PBS for 3 h. When negative result was obtained, the cultivation time was increased up to 10 days. Microscopic studies of the infected cell monolayer were performed with the Axio Scope A1 microscope (Carl Zeiss). A culture of non-infected cells was used as a negative control.

### Visualization of Cytoskeleton Rearrangements and Bacterial Cells in Infected shKEC Cells

The shKEC cells were streaked on cover glasses at a concentration of 10,000 cells/cover glass and incubated in the MEM medium with 10% FBS for 18 h. Infection was performed as described above. The negative control was the non-infected cells. After 8-h incubation, the cells were washed two times in PBS and fixed with 3.7% formalin for 10 min. The cells were then permeabilized with 0.1% Triton X-100 (Panreac, Barcelona, Catalonia, Spain) for 10 min and stained with *L. monocytogenes* antibody-fluorescein isothiocyanate conjugate (FITC; LifeSpan BioSciences, Seattle, WA, United States) Inc. for 1 h. Then cells washed three times with PBS and stained with Phalloidin Alexa Fluor 555 (Thermo FisherScientific, Waltham, MA, United States) for 20 min as described by the producer. All samples were assessed and captured thrice with Axio Scope A1 fluorescence microscope at 1,000× magnification.

### Sequence Analysis

The sequences of proteins were compared with those available in GenBank using Basic Local Alignments Tool (BLAST) analysis. For c-Met sequence were used accession numbers NP_001104541.1, NM_000245.4, NP_032617.2, and XP_003475185.1. Sequences were proofread and assembled in Unipro UGENE version 35.^1^ Protein alignment was performed using *Clustal W*. To assess the matching of the distance between multiple sequence alignments, we have utilized Unipro UGENE software. The evolutionary history was inferred by Maximum Likelihood method based on the Jones–Taylor–Thornton (JTT+G) substitution model (500 bootstrap cycles; Jones et al., 1992). Dendrograms were constructed with Mega 7.0^2^ by the method proposed by Kumar et al. (2016). Allelic numbers of InlB were determined using the *L. monocytogenes* MLST database.^3^

### Immunoblotting

The restoration of InlB expression was checked by sodium dodecyl sulfate-polyacrylamide gel electrophoresis (SDS–PAGE). Membrane-bound protein samples were prepared from overnight *L. monocytogenes* cultures grown in BHI broth supplemented with 0.2% charcoal to activate the PrfA regulon as described previously (Ermolaeva et al., 2004). SDS–PAGE was performed in accordance with the generally accepted techniques. Cell lysates of *L. monocytogenes* were boiled for 10 min, separated on 10% SDS–PAGE and transferred onto PVDF membrane. InlB was visualized with polyclonal primary anti-InlB antibodies were obtained as described earlier (Povolyaeva et al., 2020) and donkey anti-rabbit IgG HRP-labeled antibodies (Abcam, London, United Kingdom).

### Statistics

All experiments were performed in duplicate or triplicate and repeated at least three times. Student’s unpaired *t*-test, included in the Excel software package (Microsoft, Redmond, WA, United States), was used, and *p*-values of less than 0.05 were considered statistically significant.

## Results

### Sheep Cell Culture and Its Characteristics

**Figure 1:**
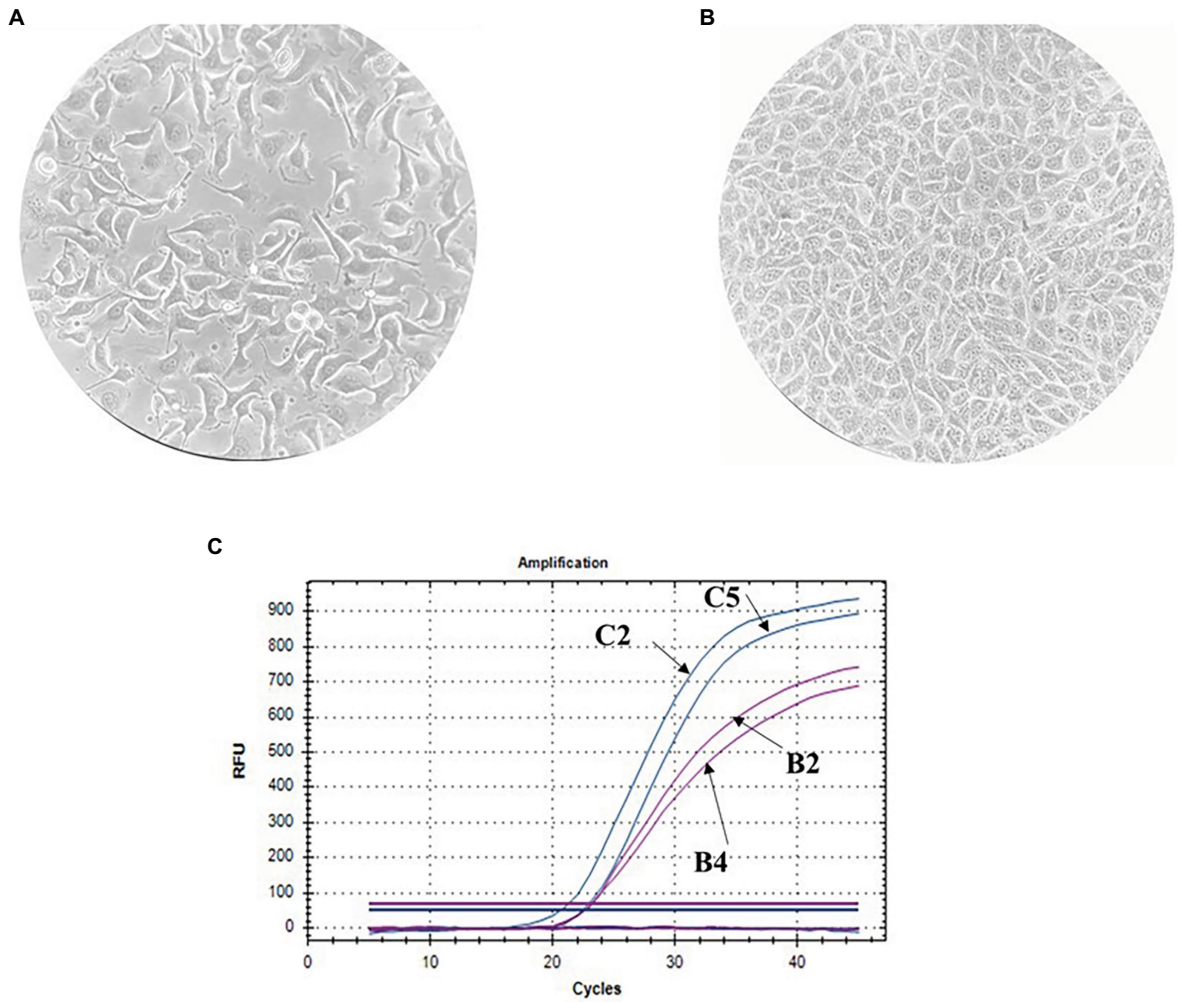
Ovine kidney cell culture (100× magnification) and invasion assay. **(A)** Propagated cell clusters. **(B)** Confluent proliferated monolayer. **(C)** Determination of the cell species by qPCR curves: B2-endogenous control in the sheep kidney cell line (shKEC) cell line, B4-endogenous control in sheep blood, C2-cytb in the shKEC cell line, and C5-cytb in sheep blood.

The finite cell shKEC used in this study was obtained and supported at the Center of Virology and Microbiology (see Materials and Methods). Morphological research of cell cultures showed that the monolayer consisted of polygonal cells belonging to the epithelial-like type (Figure 1A). The cell nuclei were oval and each contained 1–3 spherical nucleoli varying in size. The nuclear matrix was uniform. After the cells were reseeded, islet growth was observed 24 h after reseeding, and a confluent monolayer of cells was formed 48 h after reseeding. Within 3–4 days, shKEC cells formed a monolayer that persisted for 10–12 days without the medium being changed (Figure 1B). Upon the formation of a confluent cell monolayer, subsequent replating steps were performed 2–3 times a week. At a seeding concentration of 1.2 × 10^5^ cells/ml, the formation of a confluent monolayer took 48 h, and the proliferation index was 4.5. The cell monolayer showed no signs of cell degeneration, and the cytopathic effect was preserved without changing the medium for 20 days (observation period). When passaging continued, the cell monolayer retained its characteristic morphology. The viability of cells before cryopreservation was 95%. shKEC cells were stable and able to survive under conditions of low-temperature storage for more than a year. The species identity of the shKEC cells was supported by the qPCR method with the cytochrome b gene used as a target (Figure 1C). Previously, a test was conducted for the absence of cross-contamination with cells of other animals (Tsymbalova, 2012).

### *Listeria monocytogenes* Invades and Effectively Reproduces in an Ovine Kidney Cell Line

**Figure 2:**
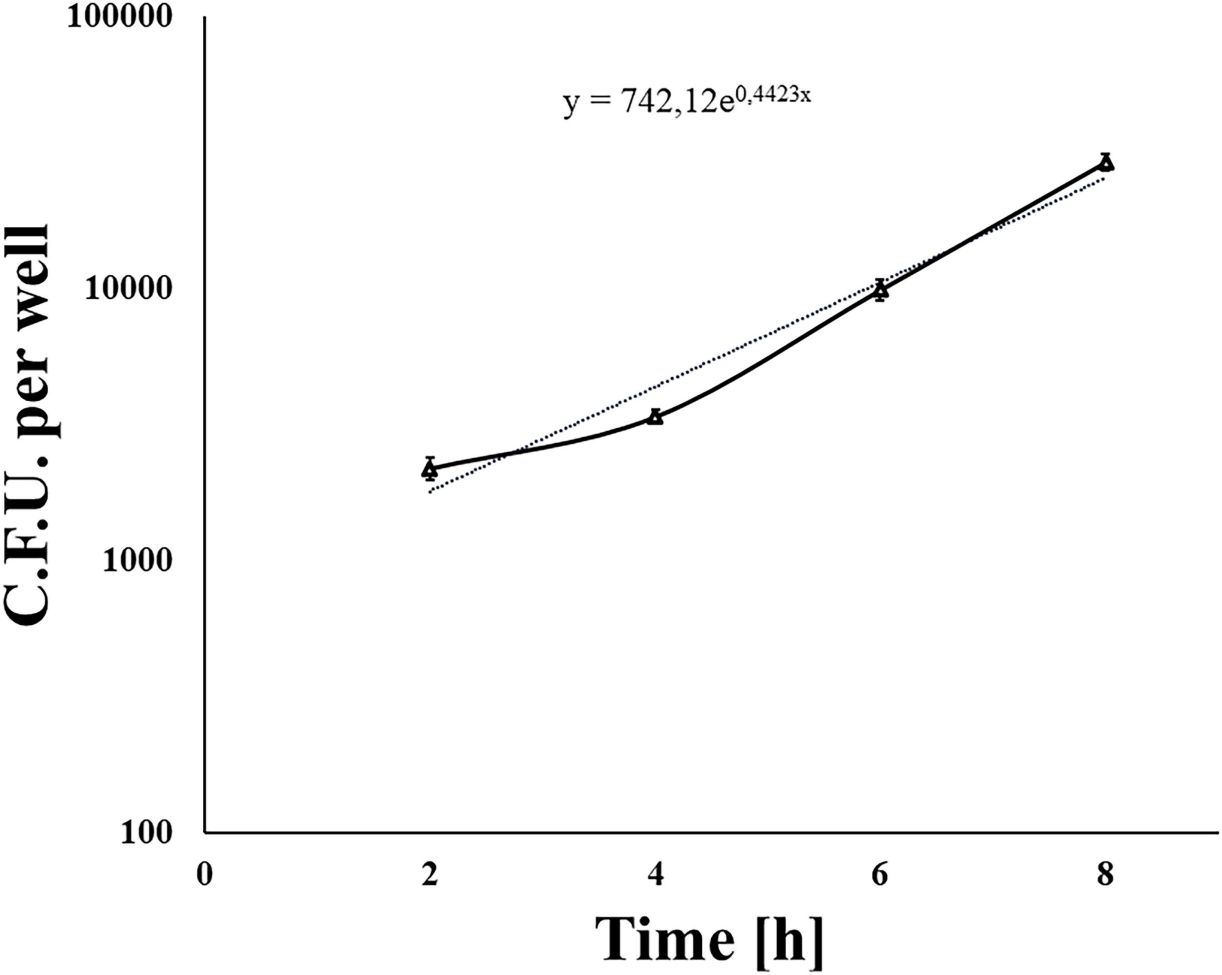
The nondotted line indicates *Listeria monocytogenes* invading and reproducing in ovine kidney cells. Approximately 1 × 10^5^ shKEC cells were infected with a multiplicity of infection (MOI) of 100, and the numbers of intracellular bacterial cells were determined 2, 4, 6, and 8 h after infection. Mean ± SD from three experiments conducted in triplicate are shown. The dotted line is the trend line.

We used a standard gentamicin assay to test *L. monocytogenes* invasion efficiency. The 70% shKEC monolayer was infected with the fully virulent laboratory *L. monocytogenes* strain EGDe at a MOI of 1:100 (cell/bacteria). Around 1 h after adding gentamicin and 2 h after infection, the average number of intracellular bacteria was 2,420 ± 599 CFU per well. The invasion efficiency, which was measured as the percentage of intracellular bacteria relative to applied bacteria, was 0.015 ± 0.004%. Next, we tested the ability of *L. monocytogenes* to multiply in the shKEC. Intracellular bacteria were counted by plating 2, 4, 6, and 8 h after infection. *Listeria monocytogenes* demonstrated exponential growth, so the number of intracellular bacteria increased more than 12-fold within 8 h after infection (Figure 2). The doubling time was 94 ± 2.5 min. Thus, *L. monocytogenes* multiplied effectively in the shKEC, with a doubling time of approximately 90 min.

### *Listeria monocytogenes* Intracellular Motion and Cell-to-Cell Spread Include Actin Polymerization Mechanisms

**Figure 3:**
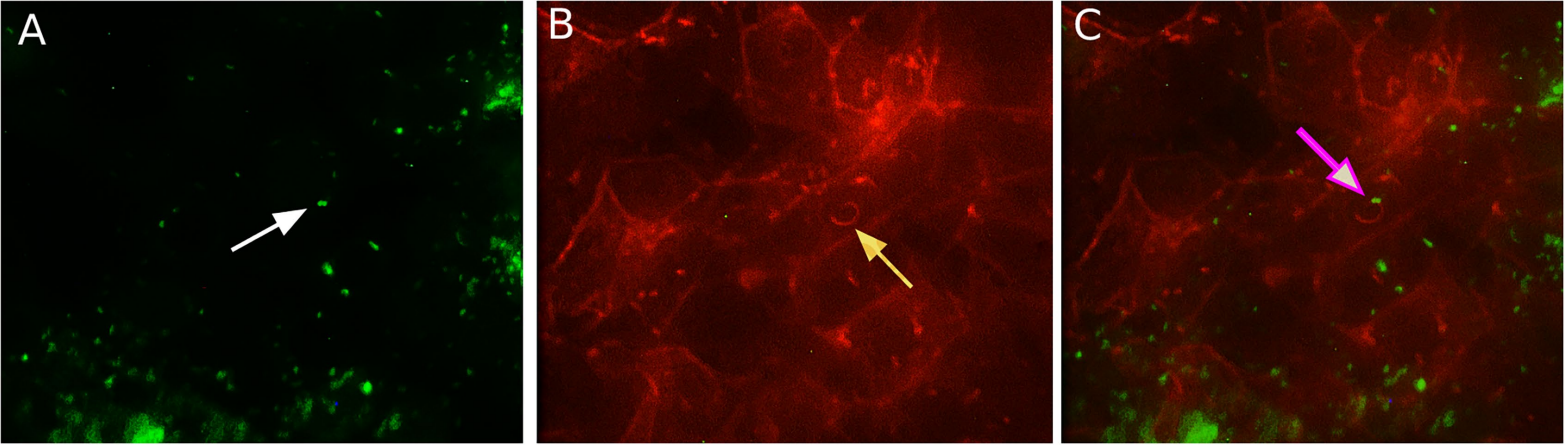
The association of *Listeria monocytogenes* with F-actin in shKEC cells. **(A)** *Listeria monocytogenes* conjugated with antibodies in the shKEC cells; the white arrow points to a bacterial cell. **(B)** Cytoskeleton of shKEC cells infected with *L. monocytogenes* and F-actin labeled with phalloidin; the yellow arrow points to a cytoskeleton rearrangement. **(C)** This part combines **(A,B)**. The pink arrow indicates the area of actin polymerization associated with bacteria cells. The samples were captured with an Axio Scope A1 fluorescence microscope at 1,000× magnification in three independent replications.

It was shown in different types of mainly human and mouse cells that *L. monocytogenes* uses host actin polymerization for intracellular movement and cell-to-cell spread (Tilney and Portnoy, 1989; Dabiri et al., 1990; Mounier et al., 1990). To analyze the activity of the actin polymerization mechanism in ovine cells, we visualized intracellular bacteria and actin microfilaments with fluorescence microscopy 8 h after infection (Figure 3). “Comet-tail” actin structures associated with bacteria were observed in infected shKEC cells. Such structures were described to provide intracellular motion, thus suggesting that bacteria used actin polymerization mechanisms.

**Figure 4:**
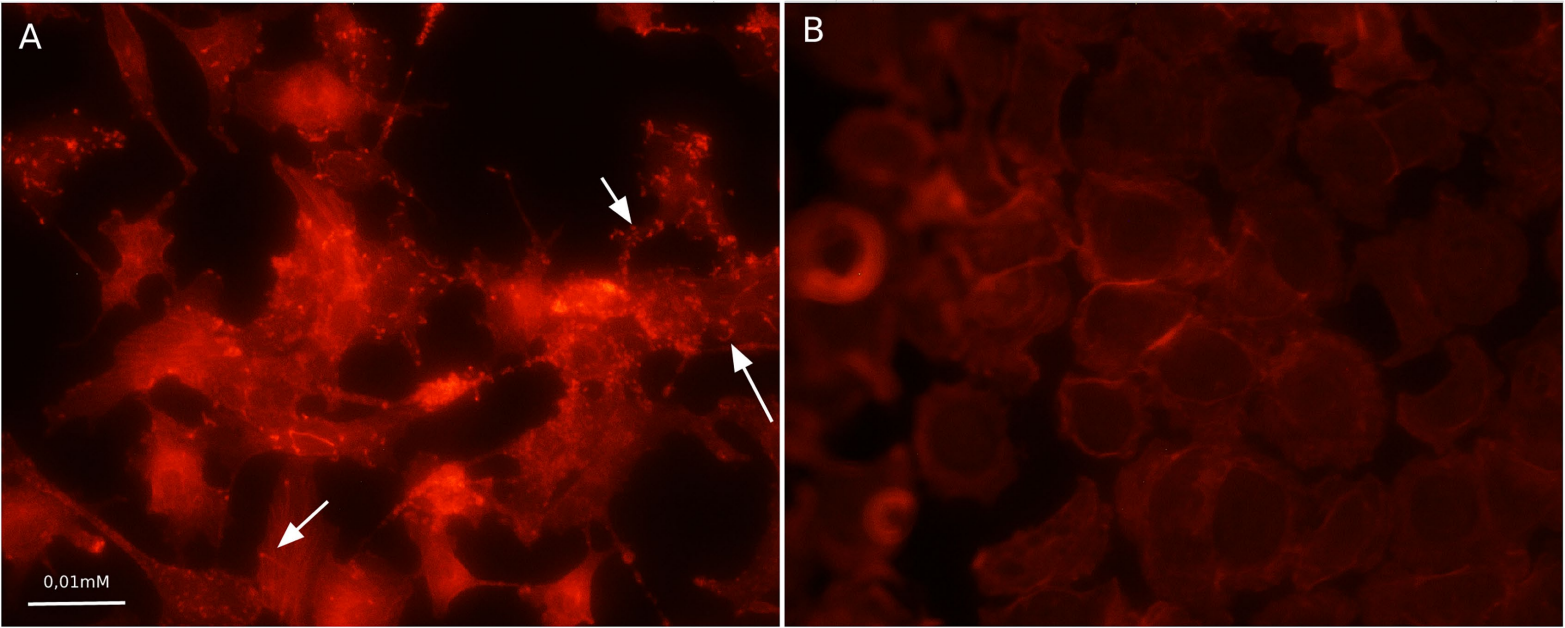
Association with F-actin in *Listeria monocytogenes*-infected shKEC cells. **(A)** shKEC cells infected with *L. monocytogenes*. The white arrows point to a cytoskeletal rearrangement stained phalloidin. **(B)** shKEC cells noninfected with *L. monocytogenes*; these cells are used as a control. The samples were captured with an Axio Scope A1 fluorescence microscope at 1,000× magnification in 10 independent replications.

Compared to the total actin cytoskeleton in the noninfected control, the total actin cytoskeleton in shKEC cells subjected to *L. monocytogenes* infection was noticeably changed (Figure 4). The observed distinctions included the appearance of not only comet-like intracellular structures but also extracellular protrusions and changes in the cell form from polygonal to nonstructured, thus suggesting a cell response to bacterial products.

**Figure 5:**
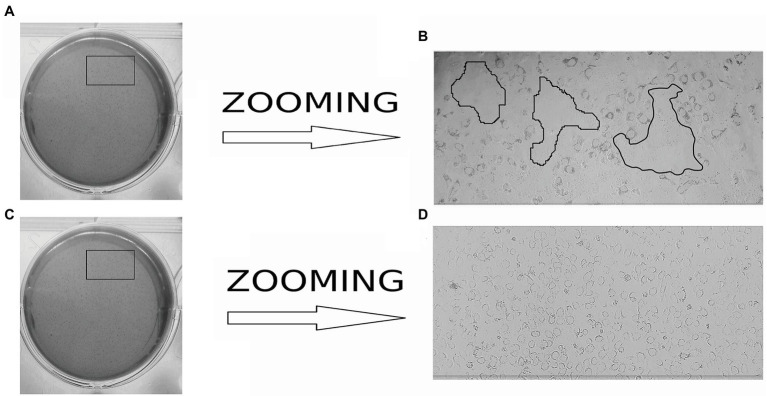
Microscopy of *Listeria monocytogenes* in a monolayer of sheep kidney epithelial cells. **(A)** Infected monolayers of shKEC cells on a six-well plate. **(B)** The enlarged image area marked in a black frame in **(A)** is the lysis of eukaryotic cells infected with *L. monocytogenes* EGDe in a monolayer of shKEC cells for 7 days on a six-well plate. **(C)** Noninfected monolayers of shKEC cells on a six-well plate. **(D)** The enlarged image area marked with areas in a black frame in **(C)** is a monolayer of uninfected cells used as a control.

Actin tail and protrusion formation are known to be involved in *L. monocytogenes* cell-to-cell spreading (Tilney and Portnoy, 1989; Dabiri et al., 1990; Mounier et al., 1990). Incubation of the infected monolayer for 24 h resulted in the death of groups of neighboring cells infected due to cell-to-cell spreading (Tilney and Portnoy, 1989). To visualize the death of neighboring host cells, we applied a standard macroscopic plaque assay using neutral red solution staining (Sun et al., 1990). Nevertheless, although this approach worked well in different cell models, including mouse L2 cell fibroblasts, human intestinal HT-29 cells, and bat kidney epithelial cells (Sun et al., 1990; Roche et al., 2001; Povolyaeva et al., 2020), the standard assay did not reveal visible plaques in the shKEC monolayer even after 10 days of incubation (data not shown). To resolve the question of whether *L. monocytogenes* could move from cell to cell, we studied infected shKEC monolayers by using light microscopy. On the 7th day after infection, we observed microscopic plaques formed by several tens of dead cells (Figure 5). Monolayers of shKEC cells were placed in the wells of six-well plates (Figures 5A,C). The formation of microscopic plaques was observed after 7 days in wells with cells infected with *L. monocytogenes* (Figure 5B). The noninfected control was intact after 7 days (Figure 5D). Therefore, *L. monocytogenes* moves intracellularly and from cell to cell in shKEC cells by using the same actin polymerization mechanisms used in human and other mammalian cells. Nevertheless, the effectiveness of cell-to-cell spreading was relatively low in shKEC cells.

### InlB Is Required for *Listeria monocytogenes* Invasion Into Ovine Cells

**Figure 6:**
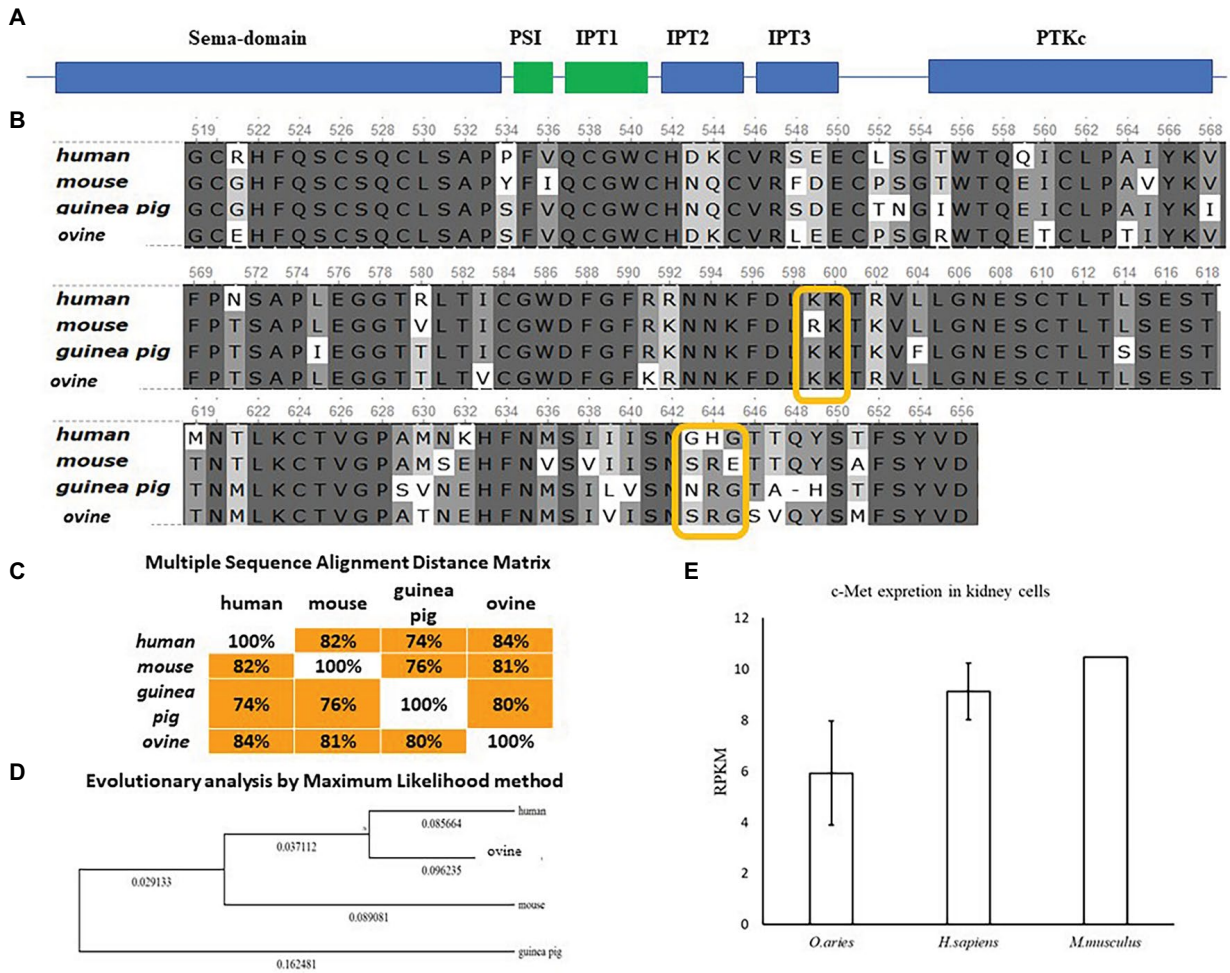
Comparative analysis of c-Met in mammals of different orders. **(A)** Schematic representation of the c-Met structure. **(B)** Alignment of protein sequences from human, mouse, guinea pig, and ovine c-Met PSI-Ig1 domains (amino acids 519–659 of human c-Met) performed using the Clustal W program (Conway Institute UCD Dublin by Des Higgins, Dublin, Leinster, Ireland). The areas marked in orange are amino acids that are necessary for the interaction between c-Met and InlB. **(C)** To assess the matching of the distance between multiple sequence alignments, we utilized UniPro UGENE software. **(D)** The amino acid sequences were analyzed using the maximum likelihood method in MEGA 7.0 by using the Jones–Taylor–Thornton (JTT+G) model and with a bootstrapping of 500 replicates. The analysis involved five sequences of the c-Met PSI-Ig1 domains. The numbers at nodes represent bootstrap values. Branch lengths are scaled according to the numbers of amino acid substitutions per site. **(E)** Data on the presence of the c-MET receptor on *Ovis aries* (PRJEB6169), *Homo sapiens* (PRJEB4337), and *Mus musculus* (PRJNA215099) kidney epithelial cells were taken from the publicly available BioProject database.

InlB is one of major invasion factors that provides *L. monocytogenes* invasion into epithelial cells *via* interactions with its target receptor c-Met (Bierne and Cossart, 2002). InlB was shown to provide species-specific interactions of *L. monocytogenes* with the guinea pig, rabbit, and bat cells (Khelef et al., 2006; Povolyaeva et al., 2020). The diagram of the structure of c-Met with the indication of the main domains is shown (Figure 6A). Structure of the human receptor tyrosine kinase Met in complex with the *L. monocytogenes* invasion protein InlB was described by Niemann et al. (2007).^4^ The partial sequence of ovine c-Met aligned with corresponding parts of human, guinea pig, and mouse c-Met proteins showed the presence of conserved lysines Lys599, Lys600, and glycine Gly645, which are involved in the interactions of c-Met and InlB (Figure 6B). The guinea pig, mouse, and ovine c-Met showed the absence of Gly643, which is present in the human protein. However, the multiple sequence alignment distance matrix method (Figure 6C) suggested that human and sheep receptors have the greatest similarity; this suggestion was supported by the maximum likelihood dendrogram, which demonstrated that the ovine InlB-binding c-Met domain formed the cluster together with human protein (Figure 6D). Generally, c-Met is produced by most mammalian epithelial cells. The BioProject database, which includes quantitative data on the presence of the c-MET receptor on different cell types, suggested that sheep kidney epithelial cells carried the c-Met receptor on their surface in amounts comparable to those on human and mouse cells (Figure 6E). Taken together, these results showed that ovine c-Met carries key amino acid residues and seemed to be able to interact with InlB.

**Figure 7:**
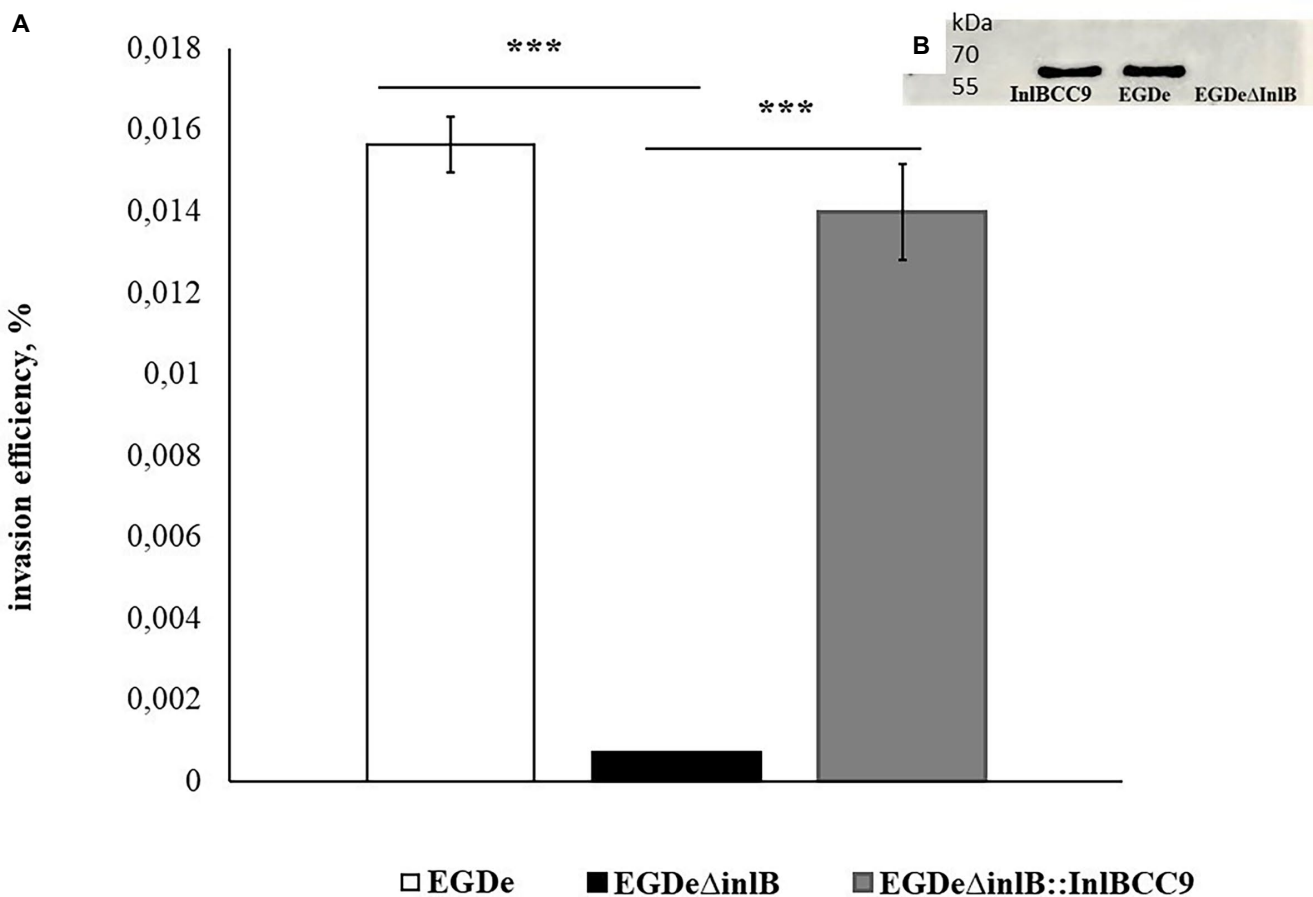
InlB is required for invasion into ruminant cells. **(A)** Invasion efficiency test in sheep kidney cells by the wild-type strain EGDe, its derivative EGDeΔinlB lacking the inlB gene and the complemented strain EGDeΔinlB::InlBCC9. The mean ± SD from three independent experiments is shown. The statistical significance of competition experiments relative to a corresponding positive control is shown (^***^p < 0.001). **(B)** Immunoblotting analysis of membrane-bound proteins of the EGDeΔinlB::InlBCC9, EGDe, and EGDeΔinlB strains. InlB was visualized with polyclonal primary anti-InlB antibodies were obtained as described earlier (Povolyaeva et al., 2020). GAPDH of *Listeria monocytogenes* was used as a control.

To further analyze InlB involvement in sheep cell infection, we compared the invasion of the wild-type strain EGDe and its derivative EGDeΔinlB lacking the *inlB* gene. The invasion efficiency of strain EGDeΔinlB was 6 × 10^−4^%, i.e., approximately 24 times less than that of the wild-type strain EGDe (Figure 7). In fact, single bacteria of the strain lacking InlB were plated from infected shKEC cell lysates, thus suggesting that InlB is strictly required for shKEC cell infection with *L. monocytogenes*.

### InlB Isoforms Characteristic of High and Low Virulence for Ruminant *Listeria monocytogenes* Clonal Complexes Differ in Their Ability to Support Invasion Into shKEC Cells

**Figure 8:**
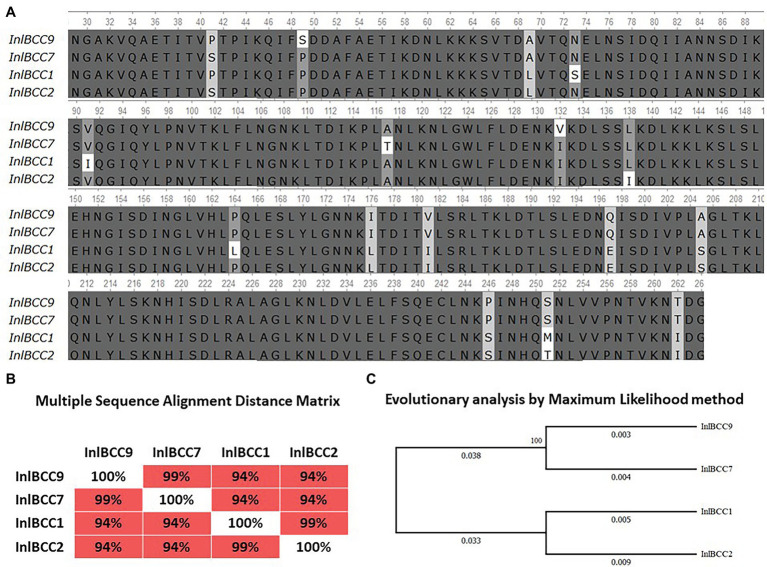
Divergence of naturally occurring variants of the InlB internalin domain. **(A)** Alignment of protein sequences InlBCC1, InlBCC2, InlBCC7, and InlBCC9 (amino acids 29-262 N-cap, LRR, and Ig-like domains) by using the Clustal W program (Conway Institute UCD Dublin, Des Higgins, Dublin, Leinster, Ireland). The areas marked in orange represent amino acids under positive selection, as shown by Tsai et al. (2006). **(B)** To assess the matching of the distance between multiple sequence alignments, we utilized UniPro UGENE software. **(C)** The amino acid sequences were analyzed using the maximum likelihood method based on the Jones–Taylor–Thornton (JTT+G) model in MEGA 7.0 and with a bootstrapping of 500 replicates. The analysis involved four sequences of the InlB complete protein. The numbers at nodes represent bootstrap values. Branch lengths are scaled according to the numbers of amino acid substitutions per site.

Previously, we showed that phylogenetically defined InlB isoforms, when placed in the same genetic background, possess a distinct potential to support *L. monocytogenes* infection into mouse and bat cells (Sobyanin et al., 2017a; Povolyaeva et al., 2020). We performed a comparative analysis of the invasion efficiencies of isogenic strains carrying distinct InlB isoforms. The InlB isoforms characteristic of strains of phylogenetically distant clonal complexes CC1, CC2, CC7, and CC9 were compared (Figure 8A). CC1 and CC7 strains were shown to be related with multiple cases of listeriosis among ruminants worldwide, while CC2 and CC9 strains, although fully virulent, are rare among animal isolates (Oevermann et al., 2010; Papić et al., 2019).

We started with an *in silico* analysis of the c-Met interacting InlB internalin domain InlB321, which comprises the cap and the LRR and the IR region (amino acids 36–321; Ferraris et al., 2010). In addition to the internalin domain, the 70-amino-acid B-repeat affecting InlB-dependent activation of c-Met-dependent Erk1/2 kinase (Copp et al., 2003; Ebbes et al., 2011) was included in the analysis (Figure 8A). The CC1- and CC2-specific isoforms differed from CC7 and CC9 by seven substitutions; this result accorded with the phylogenetic distance of the clonal complexes: CC1 and CC2 belong to phylogenetic lineage I, and CC7 and CC9 belong to phylogenetic lineage II (Figures 8B,C; Ragon et al., 2008). InlB isoforms specific for highly virulent ruminant CC1 and CC7 did not share any substitutions absent from CC2 and CC9 isoforms specific for less virulent ruminant strains.

**Figure 9:**
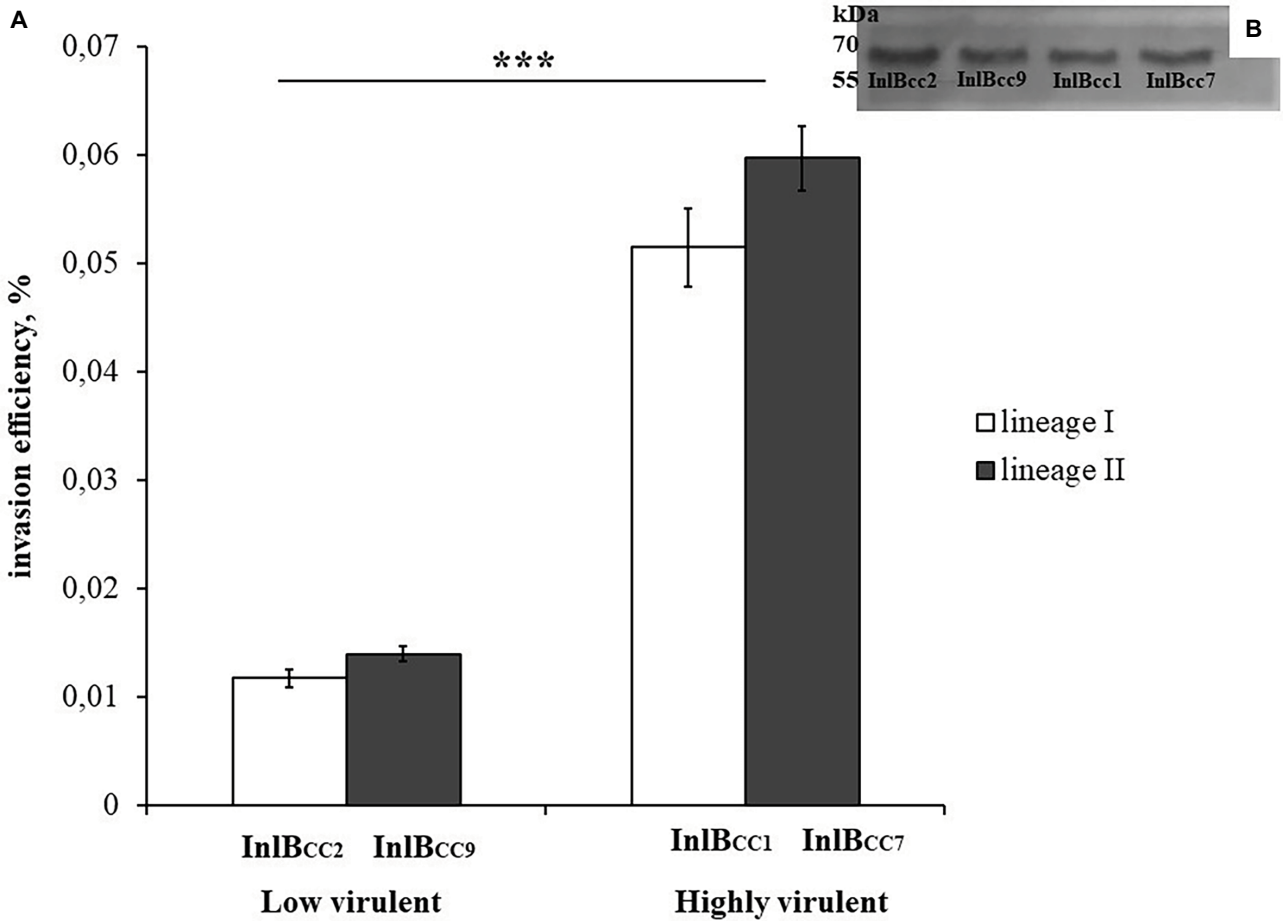
The level of invasion of *Listeria monocytogenes* into eukaryotic cells depends on the allelic variant of inlB. The recombinant strains differed only in the natural variant of internalin B. **(A)** Strains expressing natural variants of inlB characteristic of clonal complexes with low virulence reliably showed a level of invasion three times lower than the level of invasion shown by strains expressing natural variants characteristic of hypervirulent clonal complexes. The mean ± SD from three independent experiments is shown. The statistical significance of competition experiments relative to a corresponding positive control is shown (^***^p < 0.001). **(B)** Immunoblotting analysis of membrane-bound proteins of recombinant strains encoding InlB variants. InlB was visualized with polyclonal primary anti-InlB antibodies were obtained as described earlier (Povolyaeva et al., 2020). GAPDH of *L. monocytogenes* was used as a control.

To test the effect of phylogenetically defined InlB isoforms on *L. monocytogenes* interactions with sheep cells, we compared the invasion efficiencies of isogenic recombinant *L. monocytogenes* strains that differed by InlB only. The strains were previously constructed using the strain EGDeΔinlB (Sobyanin et al., 2017b). They expressed plasmid-encoded distinct InlB internalin and B-repeat domains flanked by the signaling peptide and C-end GW domains derived for strain EGDe. Distinct domains were taken from *L. monocytogenes* clones with different virulence potentials toward ruminants; these clones included the highly virulent ruminant clonal complexes CC1 and CC7 (the recombinant proteins were designated InlB_CC1_ and InlB_CC7_, respectively) and low-virulence clonal complexes CC2 and CC9 (InlB_CC2_ and InlB_CC9_, respectively). The invasion efficiency of the strains expressing InlB_CC1_ and InlB_CC7_ was at least 4,3-fold higher than the invasion efficiency of the strain expressing InlB_CC9_ (*p* < 0.0001). The strain expressing InlB_CC2_ demonstrated an invasion efficiency similar to the invasion efficiency of the strain expressing InlB_CC9_ (Figure 9). Therefore, InlB isoforms characteristic of highly virulent and low-virulence ruminant *L. monocytogenes* clonal complexes differed in their ability to restore invasion of the strain EGDeΔinlB, thus suggesting a role of naturally occurring InlB variability in the tropism of certain *L. monocytogenes* clones toward small ruminants.

## Discussion

The key stage of listerial infection is intracellular growth in nonprofessional phagocytes (Lecuit, 2020). Therefore, factors that improve active invasion or intracellular multiplication in cells of a certain mammalian species will increase the susceptibility of this species to *L. monocytogenes* infection. In this study, we demonstrated that sheep kidney epithelial shKEC cells provided favorable conditions for *L. monocytogenes* intracellular multiplication. The doubling time in ovine cells was 94 min. In Madin–Darby canine kidney epithelial cells, the doubling time reaches 180 min (Ortega et al., 2019). In kidney cells of the bat *Pipistrellus nathusii*, the doubling time of the *L. monocytogenes* strain EGDe was approximately 70 min (Povolyaeva et al., 2020). Therefore, once *L. monocytogenes* enters sheep cells, it multiplies effectively; this multiplication might be one of the factors that improves *L. monocytogenes* tropism toward sheep.

Furthermore, we demonstrated that *L. monocytogenes* used the actin polymerization mechanism described in detail in other models for intracellular and cell-to-cell movement in shKEC cells (see Figures 3, 4). Nevertheless, plaques formed by dead cells in the shKEC confluent monolayer had a microscopic character even after 7 days of incubation, thus suggesting inefficient cell-to-cell spreading. In contrast, in human HT-29 cells, microscopic plaques were observed 24 h after infection with strain EGD (Roche et al., 2001). Plaques formed in mouse L2 fibroblasts have macroscopic sizes 3 days after infection (Sun et al., 1990). What is a mechanism that restricts *L. monocytogenes* cell-to-cell spreading in ovine cells and how it affects infection in sheep has to be established in the future.

Similarly, the EGDe strain’s invasion efficiency, which reached 0.015%, was relatively low in shKEC cells. Rupp et al. (2017) observed a similar efficiency for EGDe invasion into the human epithelial colorectal adenocarcinoma cell line Caco-2, while hypervirulent strains demonstrated hyperinvasion. The EGDe strain, although fully virulent in mice, belongs to the clonal complex CC9, which is rare in both ruminant and human diseases (Oevermann et al., 2010; Charlier et al., 2017).

To elucidate the mechanisms underlying the low efficiency of the invasion of the virulent EGDe strain into sheep cells, we studied the role of the invasion factor InlB and its natural isoforms found in low-virulence and hypervirulent clonal complexes. Among the internalin family, InlB is one of two major factors in the *L. monocytogenes* invasion into epithelial cells (Pizarro-Cerdá et al., 2012). Here, we demonstrated that InlB is critically important for invasion into shKEC cells. Next, we tested whether the diversity of InlB isoforms affects *L. monocytogenes* strain tropism toward sheep. To do this, we took *L. monocytogenes* isogenic recombinant strains that differed by InlB isoforms only and compared the invasion of these strains into shKEC cells. The obtained results demonstrated that InlB isoforms cloned from strains of clonal complexes CC1 and CC7 were better able than InlB isoforms cloned from CC2 and CC9 strains to support invasion into shKEC cells. These results accord with previous data that revealed a correlation between clonal complexes belonging to the strain and virulence potential toward ruminants (Oevermann et al., 2010; Dreyer et al., 2016; Maury et al., 2016; Papić et al., 2019). In particular, clonal complex CC1 strains, which belong to phylogenetic lineage I, are highly prevalent among isolates associated with CNS infections, particularly rhombencephalitis in ruminants (Dreyer et al., 2016; Maury et al., 2016). In contrast, highly virulent human CC2 strains are rare among ruminant isolates. Meanwhile, despite the fact that they belong to phylogenetic lineage I, CC1 and CC2 strains produce LB isoforms distinct in the c-Met binding domain (Adgamov et al., 2012). Interestingly, CC1- and CC2-specific isoforms of another major invasion factor, InlA, are similar in the receptor-binding domain important for InlA invasion functions (Adgamov et al., 2012). Among strains of the phylogenetic lineage II, the same InlB isoform is produced not only by CC7 but as well by a number of clonal complexes of high and intermediate virulence (CC7, CC8, CC14, CC19, CC 20, CC21, CC155, CC177, and some others), while strains of the low virulent clonal complex CC9 carry an alternative InlB isoform (Adgamov et al., 2012; Charlier et al., 2017).

The obtained results demonstrated that InlB isoforms characteristic of highly virulent ruminant clonal complexes CC1 and CC7 were significantly better able than InlB isoforms characteristic of low-virulence ruminant clonal complexes CC2 and CC9 to support invasion into shKEC cells. The obtained results suggested that certain InlB isoforms might be input into the hypervirulence of certain clonal complexes toward small ruminants.

The question about the mechanisms underlying higher virulence has risen together with data on the different virulence potentials of certain clonal groups (Oevermann et al., 2010; Dreyer et al., 2016). At least two mechanisms, which are described in pathogenic microorganisms, can be important in the evolution of highly virulent strains: the acquisition of additional virulence factors and the selection of highly adapted isoforms of major virulence factors. It seems that both mechanisms are realized in *L. monocytogenes* evolution. Strains belonging to the highly virulent clonal complex CC1 have acquired additional virulence genes, such as inlF, inlJ1, and listeriolysin S (LLS), which are absent in most other clonal groups (Balandyté et al., 2011; Moura et al., 2016). These virulence factors were shown to improve certain stages of infection, thus resulting in hypervirulence of CC1 strains (Sabet et al., 2008; Quereda et al., 2017; Ghosh et al., 2018). However, in other models, these factors do not improve *L. monocytogenes* virulence potential (Rupp et al., 2017). Highly virulent CC4 strains lack LLS but carry a pathogenicity island of six genes called LIPI-4, thereby increasing CNS invasion capacity in humanized mice that is absent from CC1 (Dreyer et al., 2016; Moura et al., 2016). These results suggest the independent evolution of hypervirulence in different clonal groups. In addition to acquisitions of additional genes, selection of certain allelic variants of key invasion factors is an additional mechanism that increases the virulence of individual clones. This mechanism is well established in viruses but is also known in pathogenic bacteria.

Allelic variation of the *Salmonella* virulence factor PagN, which participates in the colonization of enterocytes, affects the invasive properties of different *Salmonella* subspecies and serovars, thereby binding *Salmonella typhi* to human enterocytes better than *Salmonella typhimurium* does (Wu et al., 2020). The *Yersinia pseudotuberculosis* gene encoding the Rho-modifying YopE toxin differs by three SNPs in highly virulent human FESLF strains from low-virulence strains, but all three SNPs are nonsynonymous, thus suggesting their positive selection and role in strain virulence (Timchenko et al., 2016). Earlier, we demonstrated that distinct InlB isoforms differ in their ability to support invasion into mouse and bat epithelial cells and virulence in mouse intragastric and intravenous infections (Sobyanin et al., 2017a,b; Povolyaeva et al., 2020). The results obtained in this work demonstrated that InlB variations correlating with the virulence potentials of certain clones toward ruminants produced different invasions into sheep cells. The obtained results accord with the hypothesis about the role of major virulence factor variability in the evolutionary adaptation of certain clones for host specificity or differential virulence.

## Data Availability Statement

The original contributions presented in the study are included in the article/supplementary material, further inquiries can be directed to the corresponding authors.

## Author Contributions

SE: conceptualization, formal analysis, and supervision. SE and YC: methodology, data curation, and writing – review and editing. YC: software. OK, EP, and YC: validation. YC, MA, EP, OK, EK, and OP: investigation. EK: resources. YC, OK, and SE: writing – original draft preparation. OK, YC, MA, and EP: visualization. DK: project administration. All authors contributed to the article and approved the submitted version.

## Funding

The work was supported by the Federal Research Center for Virology and Microbiology for government assignment.

## Conflict of Interest

The authors declare that the research was conducted in the absence of any commercial or financial relationships that could be construed as a potential conflict of interest.

## Publisher’s Note

All claims expressed in this article are solely those of the authors and do not necessarily represent those of their affiliated organizations, or those of the publisher, the editors and the reviewers. Any product that may be evaluated in this article, or claim that may be made by its manufacturer, is not guaranteed or endorsed by the publisher.

## Footnotes

^1^ http://ugene.net/

^2^ https://www.megasoftware.net/

^3^ https://bigsdb.pasteur.fr/listeria/listeria.html

^4^ https://www.rcsb.org/structure/2UZX
